# Comparative Meta-Analysis of Tuberculosis Contact Investigation Interventions in Eleven High Burden Countries

**DOI:** 10.1371/journal.pone.0119822

**Published:** 2015-03-26

**Authors:** Lucie Blok, Suvanand Sahu, Jacob Creswell, Sandra Alba, Robert Stevens, Mirjam I. Bakker

**Affiliations:** 1 Koninklijk Instituut voor de Tropen (KIT) Health, 1090HA Amsterdam, Netherlands; 2 Stop TB Partnership, Geneva 1211, Switzerland; 3 Koninklijk Instituut voor de Tropen (KIT) Biomedical Research, 1105 AZ Amsterdam, Netherlands; 4 Mott-MacDonald, London EC4M 7RB, United Kingdom; Fondazione Bruno Kessler, ITALY

## Abstract

**Background:**

Screening of household contacts of tuberculosis (TB) patients is a recommended strategy to improve early case detection. While it has been widely implemented in low prevalence countries, the most optimal protocols for contact investigation in high prevalence, low resource settings is yet to be determined. This study evaluated contact investigation interventions in eleven lower and middle income countries and reviewed the association between context or program-related factors and the yield of cases among contacts.

**Methods:**

We reviewed data from nineteen first wave TB REACH funded projects piloting innovations to improve case detection. These nineteen had fulfilled the eligibility criteria: contact investigation implementation and complete data reporting. We performed a cross-sectional analysis of the percentage yield and case notifications for each project. Implementation strategies were delineated and the association between independent variables and yield was analyzed by fitting a random effects logistic regression.

**Findings:**

Overall, the nineteen interventions screened 139,052 household contacts, showing great heterogeneity in the percentage yield of microscopy confirmed cases (SS+), ranging from 0.1% to 6.2%). Compared to the most restrictive testing criteria (at least two weeks of cough) the aOR’s for lesser (any TB related symptom) and least (all contacts) restrictive testing criteria were 1.71 (95%CI 0.94−3.13) and 6.90 (95% CI 3.42−13.93) respectively. The aOR for inclusion of SS- and extra-pulmonary TB was 0.31 (95% CI 0.15−0.62) compared to restricting index cases to SS+ TB. Contact investigation contributed between <1% and 14% to all SS+ cases diagnosed in the intervention areas.

**Conclusions:**

This study confirms that high numbers of active TB cases can be identified through contact investigation in a variety of contexts. However, design and program implementation factors appear to influence the yield of contact investigation and its concomitant contribution to TB case detection.

## Background

It is estimated that tuberculosis affected 8.6 million people around the world in 2012, leading to 1.3 million deaths.[1] Despite successful initiation and expansion of DOTS and the Stop TB Strategy in most parts of the world, early and effective identification of TB remains a challenge. The World Health Organization (WHO) estimates that in 2012 approximately 35% of the incident cases of TB were missed.[1]

Active case finding (ACF) approaches within population groups believed to be at higher risk of developing TB are increasingly being used by TB control programs across a spectrum of settings.[2;3] Although the exact contribution of these approaches to improving case detection in different target populations is unclear, as is how best to optimize their effectiveness, [4;5] the ample evidence indicating that close contacts of people with active TB are at high risk of infection and disease justifies special attention.[6] Two meta-analyses of contact investigation studies found a pooled prevalence of active TB (all forms) among close contacts of 3.1% (95% CI 2.2–4.4) and 4.5% (95% CI 4.3–4.8), and a pooled prevalence of microbiological confirmed TB of 1.2% (95% CI 0.9–1.8) and 2.3% (95% CI 2.1–2.5) respectively.[7;8] Largely based on these findings, screening of household contacts has now been recommended by WHO in all settings.[9]

Since ACF requires additional resources and has opportunity costs, the expected yield for a chosen strategy in a given setting and the impact on overall case notification are important considerations for policy formation.[4] The systematic reviews cited above found a strong heterogeneity in percentage of contacts identified with active TB. A number of factors were identified as potentially influencing yield of screening, including contextual aspects such as background prevalence of TB or HIV infection, as well as those related to the design and implementation of the intervention. The latter might encompass timing of screening, the strategy employed to identify and trace contacts, the operative definitions for index case and contact, criteria for testing and the diagnostic tests used. While the reviews under discussion focused on yield of active or latent TB through contact investigation, evidence on how these various cited factors contribute to higher or lower yield is still lacking.[5;7;8;10] Consequently, the international guidelines on contact investigation stress the need for further research as a basis for firm operational recommendations.[9]

Since 2010, TB REACH has funded small scale projects designed to improve early TB case detection in populations with limited access to care. The strategies and outcomes of the first wave projects are described in a different publication.[11] Contact investigation among household members of people with active TB was one of the most common interventions introduced by projects during this first funding wave. The objective of this study was to evaluate and compare household contact investigation interventions and to review the association between context or program-related factors and the yield of cases among contacts.

## Methods

This study evaluated and compared interventions in nineteen projects implemented in eleven high burden countries with lower and middle income levels across Africa, Asia and the Middle East, reviewing the association between external or program design factors and the observed yield in the different settings. The interventions were initiated between October 2010 and January 2011 and involved at least one year of implementation. In total, data from 89 quarters (4 or 5 quarters per project) were included in the study.

### Data collection

**Fig 1:**
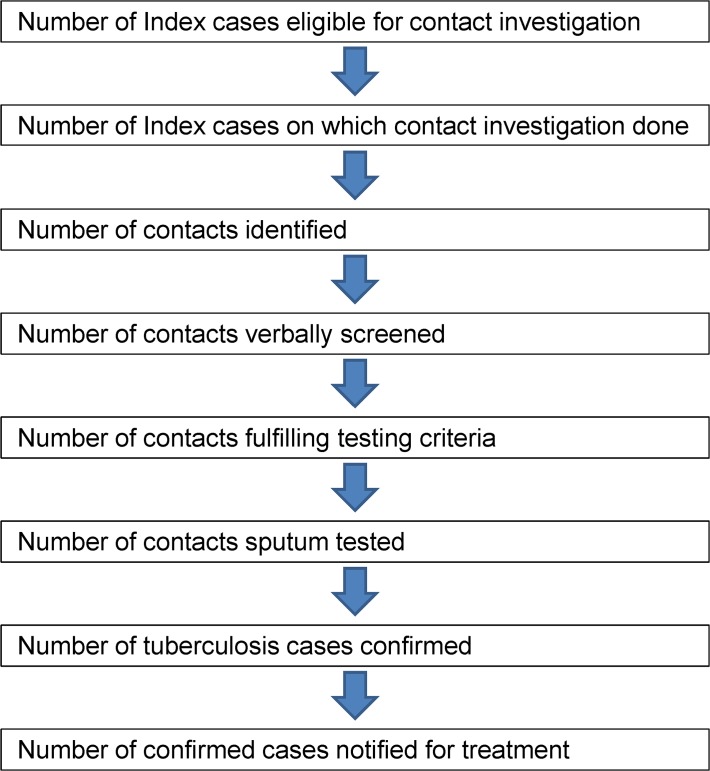
Process Indicators in Contact Investigation.

All projects reported quarterly process indicator data along with official case notification within the national TB control programs (NTP) for the administrative area in which the intervention took place. Process indicators monitored the different steps in the diagnostic pathway of contact investigation (see Fig. 1). These include: counts of index cases, household contacts screened verbally, people identified with symptoms consistent with TB, individuals tested by smear microscopy, and confirmed TB cases.

The algorithm used for identification and screening of contacts and the definitions adopted for ‘index case’, ‘contact’ and for ‘a person considered to have symptoms of TB’ were collected for each project. This information was verified through comparison with standard project visit reports by the independent monitoring and evaluation agent assigned to each grant.

### Inclusion and exclusion criteria

**Fig 2:**
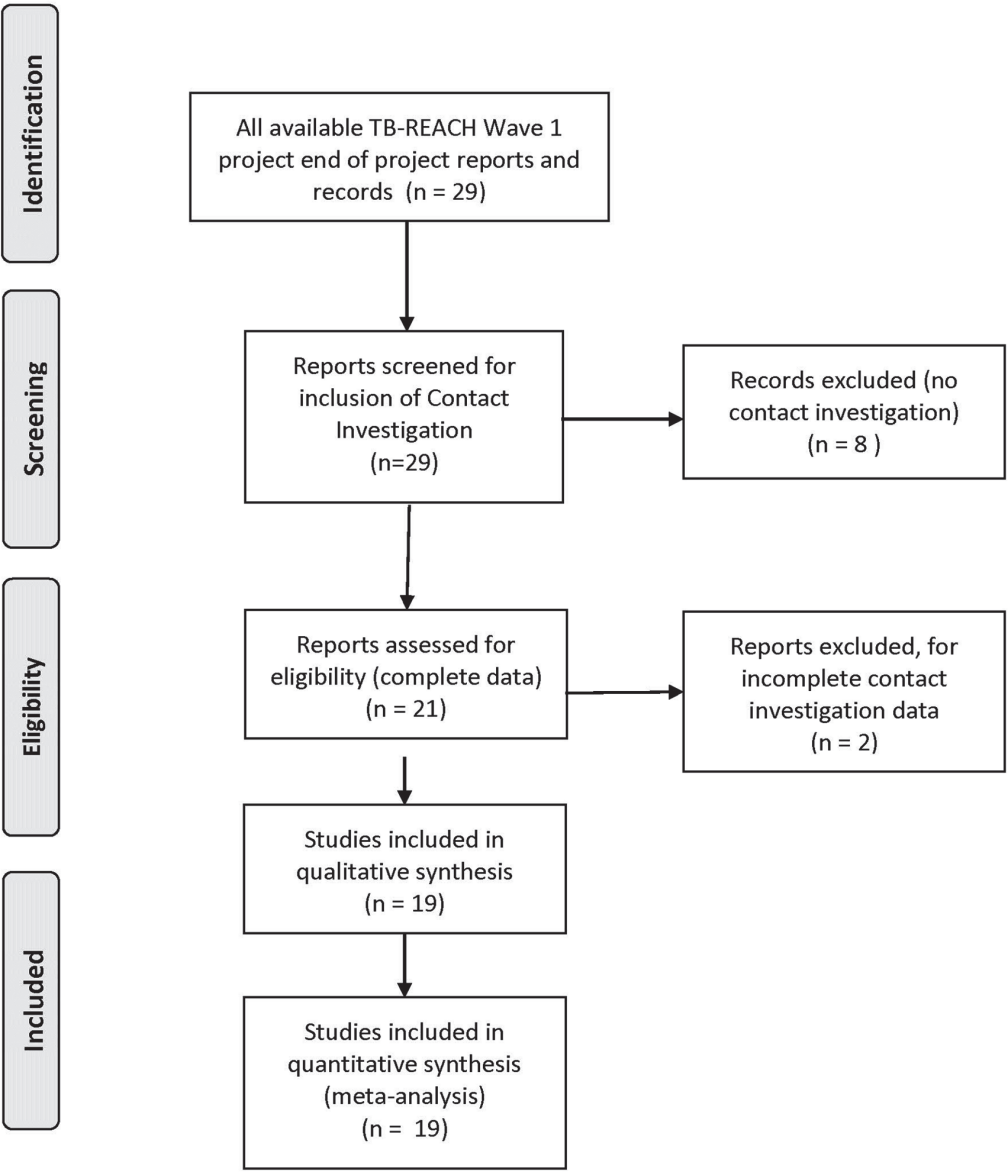
Screening for Study Inclusion Flowchart.

Projects were included if they could provide complete data on the number of contacts who were screened and on the number of contacts with a microbiological confirmed diagnosis of TB. Of twenty-one projects that implemented contact investigation, two were excluded because of incomplete data (see Fig. 2).

### Definitions

All projects used sputum smear microscopy (SSM) to diagnose TB. While one project performed both smear microscopy and culture, only the cases confirmed through smear microscopy were included in the comparative analysis of yield of sputum smear positive (SS+) cases. Nine projects also reported cases diagnosed by other means—e.g. chest X-Ray (CXR) or clinical signs—in which case the number of all forms (AF) of TB was analyzed as well.

The percentage yield of contact investigation is the primary outcome measure in our study and is defined as the proportion of all screened contacts who were diagnosed with TB times one hundred. Number needed to screen (NNS) is defined as the number of contacts needed to be screened to find one case of tuberculosis[12] and is calculated as 100 divided by the percentage yield. A secondary outcome measure is the relative contribution of contact investigation to SS+ case notification and is defined as the number of SS+ cases diagnosed through contact investigation divided by the total number of SS+ cases notified through the NTP by basic management units in which the intervention was implemented during the same time period.

Effective screening coverage of contacts is defined as the percentage of all contacts identified through interviewing index cases who are traced and have completed the full screening algorithm.

### Data analysis

All project data were entered in a Microsoft Excel database. Data were analysed following the methodology described by Hamza to conduct meta-analyses of proportions[13] implemented in Stata version 12.1 using the command xtmelogit. This method is equivalent to a random effects meta-analysis where the binomial nature of the data is taken into account and studies are thus implicitly weighed by study size.

The number of SS+ cases *Y* in each study *i* from 1 to *n* was assumed to follow a binomial distribution

$$Y_{i}∼binomial\left(m_{i},\pi_{i}\right)$$

where *m*_*i*_ is the total number of screened contacts and *π*_*i*_ is the proportion of SS+ (yield) in study *i* and the following model was fitted:

$$\log\left(\frac{\pi_{i}}{1-\pi_{i}}\right)=\alpha +\beta_{1}x_{1i}+\beta_{2}x_{2i}+\ldots +\beta_{p}x_{pi}+u_{i}$$

In this model *α*denotes the average baseline estimated SS+ log odds, *β*_*1*_ to *β*_*p*_ denote the estimated effect on the log odds of each project related independent variable (the definition used for index case, the criteria for inclusion in sputum testing, and effective coverage of contacts by screening and testing) or contextual variable (average household size, whether the intervention area was rural or urban, and estimated 2012 TB prevalence[1]) and *u*_*i*_ is a normally distributed random effects parameter which accounts for between study variation.

Given the low average baseline estimated SS+ odds (<2%) we interpret the baseline odds as a proxy for the estimated mean (pooled) yield.

First all univariate models were fitted, after which a multivariate model was built including only variables for which data were collected in all nineteen projects (complete case analysis). Household size, average number of contacts screened per index case and the coverage of contacts had to be dropped as not all projects had collected this information consistently. Subsequently we used backward elimination of variables with p>0.2. Contribution of the individual variables to the model was tested through a loglikelihood ratio test.

### Ethical approval

Contact investigation is a WHO recommended strategy and does not require ethical approval for introduction into a program. Individual projects that included additional research components in their program obtained local ethical clearance as required. Since this meta-analysis is based only on secondary analysis of aggregate and anonymous project data, it does not require additional ethical clearance.

## Results

### Implementation strategies

Table 1 provides an overview of the different contextual settings and the implementation strategies. The definition of an ‘index case’ and ‘cases considered symptomatic for TB on verbal screening’ differed by project. With two exceptions (Yemen and Nepal), projects screened only household contacts of index cases, defined as people sleeping in the same domicile and/or sharing meals with the index case for a majority of days during the previous period. The timing of screening in relation to time of diagnosis of the index case was not reported and may have varied among projects. None of the projects included repeated screening of the same household members over time.

**Table 1:** Description of intervention setting, screening approaches and definitions.

Project	Setting	Profile*			Index	type†	Test	Diagnostic test§		Screening	Screening	Incentives &
		TB	MDR	HIV			criteria‡			location|	by whom¶	enablers**
Afghanistan-1	Rural	358	3.5%	<0.1%	SS+	no child	TC-1	SSM		Home-ST	CHW	PBI
Afghanistan-2	Rural	358	3.5%	<0.1%	SS+	incl. child	TC-1	SSM	CXR	Home-ST	CHW	PBI
DRC-1	Rural	576	2.5%	1.1%	SS+	incl. child	TC-1	SSM		Home-ref / MT	HW	None
DRC-2	Rural	576	2.5%	1.1%	Incl. other	incl. child	TC-2	SSM	CXR	Home-ST	CHW	PBI for screen, transport, diagnosis
DRC-3	Rural	576	2.5%	1.1%	Incl. other	incl. child	TC-2	SSM	CXR	Home-ST	CHW	PBI for screen, transport, diagnosis
DRC-4	Rural	576	2.5%	1.1%	Incl. other	incl. child	TC-2	SSM	CXR	Home-ST	CHW	PBI for screen, transport, diagnosis
Ethiopia-1	Rural	224	1.6%	1.3%	SS+	incl. child	TC-1	SSM		Home-ref	HEWvolunteers	No
Ethiopia-2	Rural	224	1.6%	1.3%	SS+	incl. child	TC-1	SSM (few LED-FM)	CXR	Home-ST	HEW/ volunteers	PBI in form of air time for staff
Kenya-1	Mixed	299	2.5%	6.1%	SS+	no child	TC-1	SSM	CXR	Home-ST /-ref	CHW	Bus fare & lunch allowance plus PBI
Kenya-2	Urban	299	2.5%	6.1%	SS+	incl. child	TC-2	SSM		Home-ref	CHW	Small in kind support
Lao rep	Rural	514	4.9%	0.3%	SS+	no child	TC-2	SSM		Home-ST	CHW	Monetary incentives TB manager
Nepal	Mixed	241	2.3%	0.3%	SS+	incl. child	TC-2	SSM		Home-ST	NGOstaff	None
Nigeria	Rural	161	2.9%	3.1%	SS+	no child	TC-2	SSM		Home-ref	CHW	PBI
Pakistan-1	Mixed	376	3.5%	<0.1%	SS+	incl. child	TC-2	SSM		Home-ref	SW	PBI accompanied referral
Pakistan-2	Urban	376	3.5%	<0.1%	Incl. other	incl. child	TC-2	SSM	CXR	Home-ref	CHW	
Uganda-1	Rural	175	1.4%	7.2%	SS+	incl. child	TC-3	SSM		Home-ST	CHW/HW	Monetary incentive MoH-HW
Uganda-2	Rural	175	1.4%	7.2%	SS+	incl. child	TC-3	SSM		Home-ST	volunteer	PBI
Yemen	Mixed	70	1.7%	0.1%	SS+	incl. child	TC-3	LED-FM (culture)		Home-ST / invite	CHW	Transport refund contacts
Zimbabwe	Urban	433	1.9%	14.7%	SS+	incl. child	TC-2	SSM	CXR	Home-ref	HW	None

* WHO 2012: TB data[1] presented as cases per 100,000 pop/year; MDR = multi drug resistance (%) among newly diagnosed TB cases[1]; HIV prevalence(%) among adults aged 15–49 years[14]

† SS+ = smear positive only; ‘incl. other’ = includes SS- pulmonary and/or extra-pulmonary TB; ‘incl. child = children included as index case; ‘no child’ = no children as index case.

‡ TC-1 = Cough ≥ 2weeks with or without other symptoms; TC-2 = Any (or combination) out of five to seven TB related symptoms; TC-3 = All contacts irrespective of symptoms

§ SSM = Standard sputum smear microscopy; LED-FM = Light emission diode fluorescent microscopy; CXR = Chest X-ray

| Home-ref = screening at household followed by referral of suspects for testing; Home-ST = Screening and sputum collected at home and transported for testing; Invite = contacts are invited to present at facility for screening, MT = Mobile team outreach to community for screening and testing.

¶ HW = health staff in clinic or through outreach; HEW = health extension worker; CHW = community health worker; SW = social worker

** PBI = performance based monetary incentives

In all projects, household contacts of index cases were visited at home by a community- based health worker or volunteer or by a clinic staff member. Subsequently, contacts fitting testing criteria were either referred for testing or a sputum sample was taken to be tested in a nearby facility. In Yemen, where deterioration of the security situation over the course of the year made home visits unpractical, the project provided contacts with transportation costs for travel to the hospital for screening. Eleven projects offered the staff engaged in screening some level of monetary or in kind incentive. Two projects offered patient support for referral.

### Screening yield

**Fig 3:**
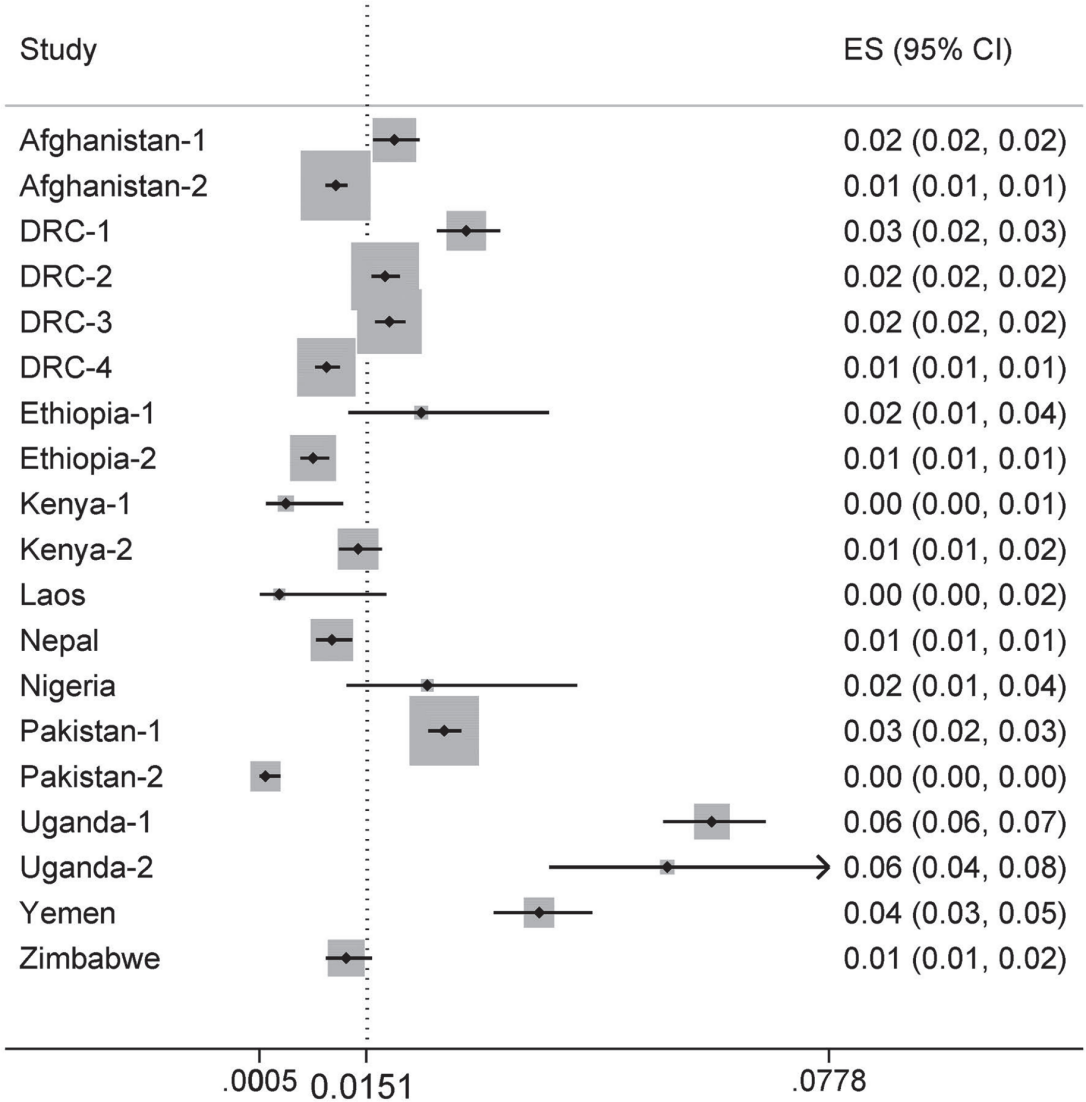
Forest plot of the proportion SS+TB among screened household contacts of TB patients.

**Fig 4:**
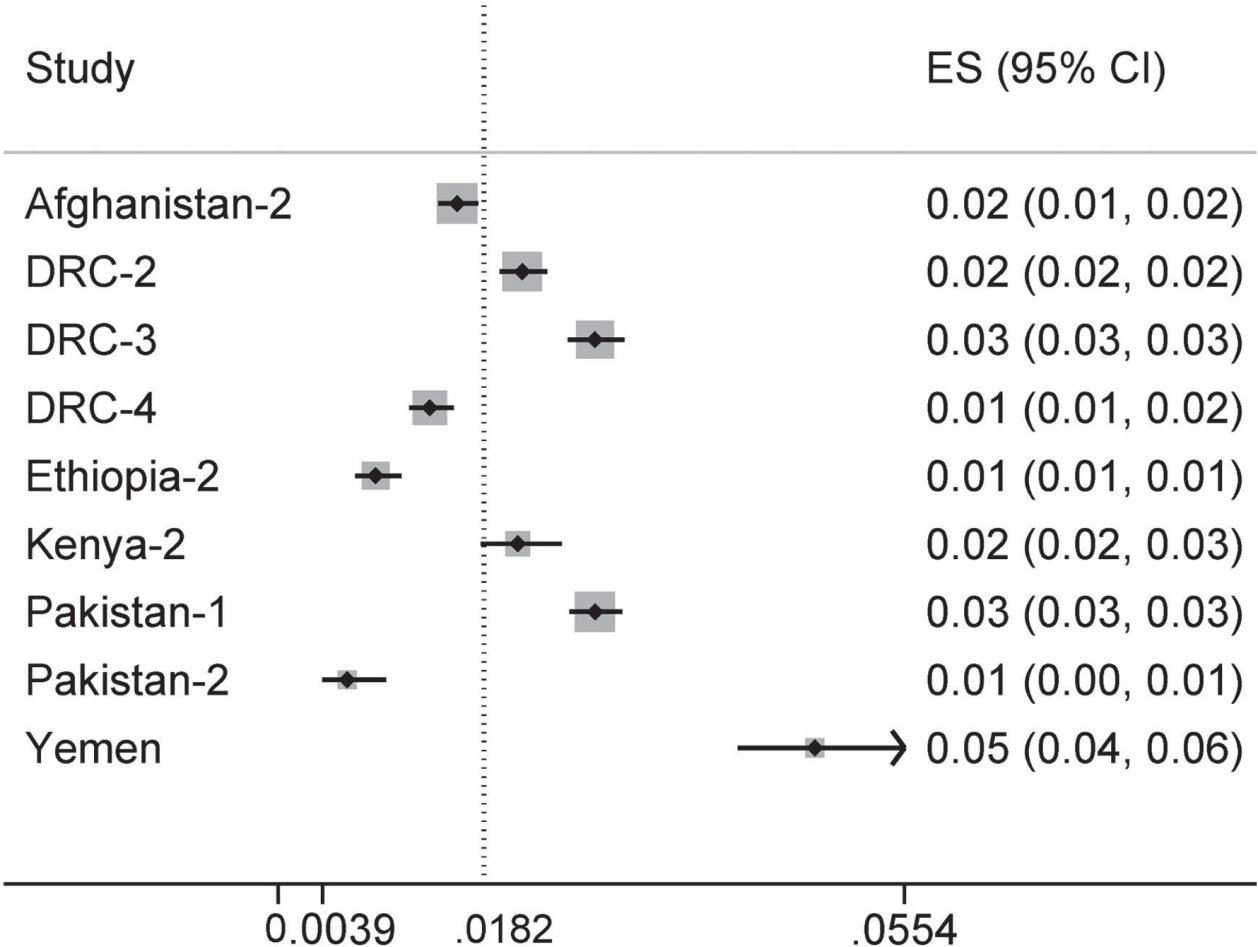
Forest plot of the proportion of any form of TB among screened household contacts of TB patients.

The nineteen projects cumulatively screened 139,052 household contacts of more than 30,000 index cases during the reporting period (S1 Table). A total of 2,498 contacts were diagnosed with SS+ TB. Pooled analysis provides a percentage yield of 1.5% (95%CI 1.0–2.2%). The percentage yield by project ranged from 0.1% to 6.2% showing substantial heterogeneity (I^2^ = 97.7%) (Table 2, Figs. 3 and 4). Nine projects also reported all forms of TB diagnosed through contact investigation, resulting in a pooled percentage yield of all forms of TB of 1.8% (95% CI 1.2–2.7%; I^2^ = 97.8%) ranging from 0.6% to 4.8%. The pooled SS+ percentage yield in this subset of projects was 1.2% (95% CI 0.7–2.1%; I^2^ = 97.9%) ranging from 0.1% to 3.8%.

**Table 2:** Number of people screened and number of smear positive (SM+) and all forms of TB identified through contact investigation in 19 projects.

Project	Contacts screened	SS+	% SS+/screen	95% CI*	NNS (SS+)	All TB	% All TB/screen	95% CI*	NNS (All TB)
Afghanistan-1	7232	136	1.9%	(1.6–2.2%)	53				
Afghanistan-2	19383	210	1.1%	(0.9–1.2%)	92	307	1.6%	(1.4–1.8%)	63
DRC-1	5814	166	2.9%	(2.4–3.3%)	35				
DRC-2	18384	322	1.8%	(1.6–1.9%)	57	397	2.2%	(1.9–2.4%)	46
DRC-3	16552	300	1.8%	(1.6–2.0%)	55	464	2.8%	(2.6–3.1%)	36
DRC-4	13265	127	1.0%	(0.8–1.1%)	104	178	1.3%	(1.1–1.5%)	75
Ethiopia-1	490	11	2.2%	(0.9–3.6%)	45				
Ethiopia-2	8005	62	0.8%	(0.6–1.0%)	129	69	0.9%	(0.7–1.1%)	116
Kenya-1	742	3	0.4%	(0.0–0.9%)	247				
Kenya-2	6274	87	1.4%	(1.1–1.7%)	72	133	2.1%	(1.8–2.5%)	47
Lao rep	316	1	0.3%	(0.0–0.9%)	316				
Nepal	6596	68	1.0%	(0.8–1.3%)	97				
Nigeria	387	9	2.3%	(0.8–3.8%)	43				
Pakistan-1	19191	490	2.6%	(2.3–2.7%)	39	538	2.8%	(2.5–3.0%)	36
Pakistan-2	3111	4	0.1%	(0.0–0.3%)	778	19	0.6%	(0.3–0.9%)	164
Uganda-1	4638	287	6.2%	(5.5–6.9%)	16				
Uganda-2	573	32	5.6%	(3.7–7.5%)	18				
Yemen	3200	123	3.8%	(3.2–4.5%)	26	152	4.8%	(4.0–5.5%)	21
Zimbabwe	4899	60	1.2%	(0.9–1.5%)	82				
Total	139052	2498				2257			
	Pooled % yield:		1.5%	(1.0–2.2%)	67 (45–100)		1.8%	(1.2–2.7%)	56(37–83)

*Confidence intervals were derived through a Wilson score interval method

The median number of people screened by household was 3.9 (IQR 2.8–5.2). Fourteen projects reported data allowing the calculation of the number of cases diagnosed per index case. For each one hundred index cases a pooled estimate of 5.1 SS+ cases were confirmed (95% CI 3.1–8.4), ranging from a minimum of 0.8 to maximum 26.6. A pooled estimate of 6.9 cases of all forms of TB were identified per hundred index cases (95% CI 4.4–10.6; min-max range 2.0–17.7).

### Factors associated with changes in yield

The most commonly used definition of an index case was ‘all SS+ cases’ (n = 11). Some projects used ‘only adult SS+ cases’ as index case (n = 4) or ‘adult pulmonary cases and any form of childhood TB’ (n = 3). One project screened contacts of index cases with ‘any form of pulmonary TB’ (n = 1). Stratification of percentage yield by different (combinations of) index cases suggests that SS+ index cases are associated with a higher percentage yield of sputum positive contacts than SS- index cases. Inclusion of other types of TB as index case reduced the odds of finding a confirmed case by a factor 0.31 (95%CI 0.15–0.62). We could not confirm an association between using a child as an index case and percentage yield and this factor was dropped from the model during backward elimination in the multivariate analysis (Table 3).

**Table 3:** Association between program design or contextual factors and the percentage yield of contact tracing using random effect, exact binomial logistic regression.

		Univariate models (complete case analysis)					Multi variate model (N = 19)		
		N projects	N screened	OR*	(95% CI)	p-value†	aOR*	(95% CI)	p-value†
**Program design and implementation**									
Definition index case									
- Index includes children	Reference	15	8677	1			dropped in backward elimination		
- Index does not include children		4	130375	0.59	(0.22–1.64)	0.317			
- SS+ index cases only	Reference	15	87740	1			1		
- SS+ and (SS- and/or EPTB) index cases		4	51312	0.49	(0.20–1.20)	0.119	**0.31**	**(0.15–0.62)**	**0.001**
Definition Suspected TB (people to be tested)		19	139052			0.005			**<0.001**
- Contact with cough at least 2 weeks	Reference	6	41666	1			1		
- Contact with any TB related symptoms		10	88975	0.87	(0.47–1.62)	0.663	1.71	(0.94–3.13)	0.080
- Any HH contact irrespective of symptoms		3	8411	**3.94**	**(1.72–9.03)**	**0.001**	**6.90**	**(3.42–13.93)**	**<0.001**
Average HH size (contacts identified per index case)		7	63090	0.72	(0.50–1.04)	0.080	not included in multivariate analysis (‡)		
									
Average number of HH contacts screened per index case		14	118533	0.77	(0.57–1.06)	0.109	not included in multivariate analysis (‡)		
Coverage identified contacts (per 10% increase) §		7	63090	0.79	(0.50–1.23)	0.293	not included in multivariate analysis (‡)		
**Context and setting**									
Background TB prevalence (unit of increase: 100/100,000 pop)		19	139052	0.84	(0.67–1.07)	0.160	1.22	(0.99–1.50)	0.057
Setting									
Rural	Reference	12	95039	1			1		
Urban/mixed		7	44013	0.58	(0.27–1.27)	0.175	**0.53**	**(0.31–0.90)**	**0.018**

*The Odds ratio compares the chance of confirming contacts as being a SS+ case in different program designs and context settings

† Wald test of significance of effect; LLR test of significance of variable in the model

‡ variable could not be included in multivariate analysis due to unavailability of data for several projects

§ Coverage of contacts defined as percentage of identified contacts that enter screening algorithm

The factor that appeared to be most strongly associated with percentage yield was the criteria used for inclusion in sputum examination. Six projects tested “persons with cough lasting at least two weeks with or without other symptoms”, ten projects used the more inclusive definition of “a contact reporting any tuberculosis related symptoms”, and three projects tested all household contacts, irrespective of having TB-related symptoms. We found a substantial increase in percentage yield for the lesser and least restrictive definitions as compared to the most restrictive definition with an aOR of 1.71 (95%CI 0.94–3.13) and 6.90 (95%CI 3.42–13.93) respectively.

Higher background TB prevalence was associated with a higher percentage yield (aOR 1.22 for each increase with 100/100,000 population; 95% CI 0.99–1.50. Projects in urban or mixed settings produced a significantly lower percentage yield than did those in rural settings (aOR = 0.53, 95%CI 0.31–0.90) (Table 3).

A sensitivity analysis, run to explore a potential bias towards the larger studies, revealed no substantially different results for the model after exclusion of the five largest studies. The significant associations shown in Table 3 remained significant with the exception of rural or urban setting, for which the p-value changed to 0.067 (refer to annex 2 for details).

Contact investigation coverage of both index cases and their contacts varied substantially. For projects with complete data, coverage of index cases ranged from 2.8% to 91.7%. The proportion of identified contacts who were traced and screened ranged from 42.9% to 100%. The proportion of symptomatic contacts who submitted a sputum sample for testing ranged from 13.6–93.4%. In seven projects with sufficiently detailed data we found an effective screening coverage of contacts ranging from 11.4–74.1% (Table 4). Larger average household size (number of contacts identified for each index case) and higher screening coverage of the identified household members were associated with a statistically insignificant lower percentage yield. We found an odds ratio of 0.72 (95%CI 0.50–1.04) for larger household size and 0.79 (95%CI 0.50–1.23) for each 10% increase in screening coverage of household members. Because this information was missing for a substantial number of projects, these variables were not included in the multivariate analysis.

**Table 4:** Coverage of contact screening (n = 15).

	Index cases			contacts			symptomatic			Full
Project	Eligible‡	CI^†^ done	% coverage	Iden-tified	Verbal screened	% coverage	Number	Tested (sputum)	% coverage	screening identified contacts
Afghanistan-2	4447	2894	65.1%		19383			6782		
DRC-2	6773	4528	66.9%	25264	18384	72.8%	3685	3428	93.0%	67.7%
DRC-3	6360	5831	91.7%	20672	16552	80.1%	2940	2719	92.5%	74.1%
DRC-4	6353	4346	68.4%	30951	13265	42.9%	1369	1236	90.3%	38.7%
Ethiopia-2	5090	3499	68.7%	8005	8005	100.0%	1949	1290	66.2%	66.2%
Kenya-1	3121	151	4.8%		742			147		
Kenya-2	12780	2860	22.4%		6274		3482	2246	64.5%	
Lao rep	2179	61	2.8%		316			26		
Nepal	4338				6596		2708	2529	93.4%	
Nigeria	2532				387		86	58	67.4%	
Pakistan-1	3608	3037	84.2%		19191		3478	2160	62.1%	
Pakistan-2	3230	481	14.9%	3704	3111	84.0%	316	43	13.6%	11.4%
Uganda-2	2041	308	15.1%	882	573	65.0%		483	84.3%	54.8%(*)
Yemen	2259	1030	45.6%	5059	3200	63.3%	1356	2093	65.4%	41.4%(*)
Zimbabwe	2346	1313	56.0%		4899			210		

(*) all contacts were planned to be tested irrespective of symptoms

^†^ CI = Contact investigation

‡ Eligible index cases are SS+ cases notified with exception of DRC(3x) and Pakistan-2 where also other forms of TB were considered as index case as index

### Contribution of contact investigation to tuberculosis case notification

Eighteen projects reported on cases found through contact investigation as well as overall TB case notification in the administrative area where the intervention took place. In these projects, out of a total of 68,751 smear positive TB cases notified during the study period 2,375 were found by contact screening. Important variations were found among the projects, ranging from less than 1% to 14.1% of all cases notified in the intervention area having been identified through contact investigation, with a pooled estimate of 1.8% (95%CI = 0.9–3.6%) (see Table 5). Not surprisingly, there was a strong association between the proportion of TB cases found through contact screening and increased coverage of index cases (OR = 1.6 for each 10% increase in coverage; 95%CI = 1.3–1.9).

**Table 5:** Yield of active case finding and proportion of notified SS+ cases found through contact investigation (n = 18).

	SS+ cases notified in population	found through contact investigation			Index coverage
Project	Cases	Cases	%	(95% CI)	%
Afghanistan-1	2382	136	5.7%	(4.8–6.6%)	
Afghanistan-2	4777	210	4.4%	(3.8–5.0%)	60.6%
DRC-1	2610	166	6.4%	(5.4–7.3%)	
DRC-2	5767	322	5.6%	(5.0–6.2%)	66.9%
DRC-3	5145	300	5.8%	(5.2–6.5%)	91.7%
DRC-4	4802	127	2.6%	(2.2–3.1%)	68.4%
Ethiopia-1	687	11	1.6%	(0.7–2.5%)	
Ethiopia-2	5090	62	1.2%	(0.9–1.5%)	68.7%
Kenya-1	3121	3	0.1%	(0.0–0.2%)	4.8%
Kenya-2	12780	87	0.7%	(0.5–0.8%)	22.4%
Lao rep	2179	1	0.0%	(0.0–0.1%)	2.8%
Nepal	4338	68	1.6%	(1.2–1.9%)	
Nigeria	2532	9	0.4%	(0.1–0.5%)	
Pakistan-1	3608	490	13.6%	(12.5–14.7%)	84.2%
Pakistan-2	1292	4	0.3%	(0.0–0.6%)	14.9%
Uganda-1	2041	287	14.1%	(12.6–15.6%)	52.8%
Uganda-2	2259	32	1.4%	(0.9–1.9%)	13.6%
Zimbabwe	2346	60	2.6%	(1.9–3.2%)	56.0%
	Pooled estimate:		1.8%	(0.9–3.5%)	

## Discussion

This analysis of nineteen contact investigation interventions represents a large combined data set of the screening of 139,052 contacts resulting in the diagnosis of 2,498 SS+ TB cases. A pooled percentage yield of SS+ TB 1.5% (95%CI 1.0–2.2%) confirms a high TB prevalence among household contacts of people with active TB, in line with current available knowledge.[7;8] While a publication bias towards studies with higher yields cannot be ruled out in systematic literature reviews, we are confident that there is no such bias in this study as we included all interventions irrespective of their results. All projects in this study are funded by TB REACH and therefore represent settings of relatively high burden and have a focus on populations with constrained access to TB services. Our findings support recent recommendations that contact investigation be conducted as a means to improve early tuberculosis case detection also in these settings.[9]

Our finding that each 100 index cases yielded on average 5.1 smear positive contacts, and 6.9 contacts with any form of TB, is likely to be an underestimate of the true number of cases for each index case. The dropout was substantial in all stages of the screening process (tracing, screening and testing) with only approximately half of all identified contacts entering and finishing full screening. Incomplete screening of household contacts may represent a screening bias towards people identifying themselves as symptomatic and at risk. Such a selection bias is particularly likely when contacts have been encouraged to attend a clinic if symptoms develop, but may be less so in situations where contacts are screened at home[15] as was the case in most of the projects included in this study. Dropout of contacts between screening and testing, on the other hand, would have led to cases being missed, resulting in a reduced percentage yield. Other studies have shown that acceptance of TB screening is generally high, though heterogeneity in screening coverage has been observed and is likely to be the result of a variety of factors.[16]

The percentage of contacts in our study who were confirmed with any form of TB was low in comparison to other studies, indicating that diagnosis of SS- and extra pulmonary forms was given less priority. Eight projects reported access to CXR for at least some part of the smear negative contacts exhibiting symptoms. In all other instances diagnosis relied on clinical judgment and trial treatment with antibiotics. This is likely to have delayed the diagnosis and these cases may not have been reported as resulting from contact investigation.

An estimated 67 (95%CI 45–100) contacts need to be screened to find one SS+ TB case; 56 (95% CI 37–83) for any type of TB. This translates to performing just over fourteen household visits. Since home visits and contact screening require extra resources and put a burden on health staff, these enhanced requirements need to be carefully balanced against expected yield.[4] Our study demonstrates a wide variation in SS+ yield, ranging from 0.1 to 6.2%. Accordingly, predicting which sub-populations of contacts will have the highest prevalence and identifying screening algorithms contributing to higher yield will be important to future guidance on contact investigation.

The WHO guidance on contact investigation does not provide specific screening protocols[9] and the current practice of contact investigation across countries shows significant heterogeneity in algorithms and definitions.[17] Our study indicates that the criteria used for asking contacts to submit a sputum sample for testing has a strong correlation with the percentage yield. Projects that tested contacts with any TB related symptoms and projects that tested all contacts regardless of having symptoms showed substantially higher percentage yield of SS+ cases than the projects using a strict criteria of ≥2 weeks cough. Community-based ACF studies in South Africa and Western Kenya[18;19] and a meta-analysis of ACF strategies in populations with high HIV prevalence[20] found substantial numbers of cases among asymptomatic people, possibly attributable to TB-HIV co-infection, and substantial numbers of asymptomatic, smear or culture positive cases have been identified in prevalence studies in various other settings as well[21;22]. A systematic review found prolonged cough to have a sensitivity of less than 25% in low HIV prevalence areas, with any symptom having a sensitivity of close to 70%[23].These findings may suggest that a lack of symptoms does not rule out early disease and, if confirmed by further research, may justify testing of all contacts irrespective of having symptoms.

Widening inclusion criteria for testing will increase the number of sputum tests to be performed. SSM is relatively cheap and considered cost effective when relatively low rates of yield are expected, particularly in comparison with algorithms using more expensive techniques.[24] The downside of the higher sensitivity of more inclusive testing criteria-testing all contacts regardless of having symptoms- is the generally lower positive predictive value of such algorithm.[22;25] Since this may lead to erroneously diagnosed cases, the use of a confirmatory test with higher specificity (culture or molecular test) might be considered if the prevalence among screened contacts is low.[4;26] To this end new guidance on Xpert MTB/RIF testing allows it to replace microscopy where resources permit.[27;28]

Although recent WHO guidance on contact investigation recommends concentrating on index cases who have SS+ pulmonary TB or MDR TB, who are infected with HIV, or are children under five with TB, evidence to support this recommendation is still limited.[9] In our study the projects that used only SS+ index cases had significantly higher percentage yields than projects that included SS- and extra-pulmonary cases. This supports prioritization of the SS+ index cases.

We found some indication of increased prevalence of SS+ TB among contacts in countries with higher background prevalence of TB. So far meta-analyses have only found a significant correlation between national TB prevalence and yield when screening HIV positive people.[20] It is possible that the effect of prolonged intensive exposure at household level outweighs the risk of exposure in the community. Since the projects in our study targeted populations with low TB service coverage, national prevalence estimates may not have been a good marker for undetected TB in the targeted populations; inaccuracies in national prevalence estimates also cannot be ruled out.

Surprisingly, we found the percentage yield of contact screening was higher in rural than in urban settings. One of the studies in Pakistan compared urban and rural results and found a similar significantly higher prevalence among rural household members than for those in urban areas.[15] The reason for this difference is not clear, although it is possible though that prevalence may have been higher due to a lower socio–economic status. Furthermore access to care in rural areas is usually more limited, resulting in fewer people spontaneously seeking care.

The contribution of contact investigation to overall case notification in the studies presented here ranged from <0.1% to 14.1% of all notified cases in the study areas. So far data on the potential contribution of contact investigation is limited.[7] Morocco reported that systematic contact investigation on all confirmed cases between 1993–2004 contributed 5.6% of all notified TB cases.[29] Contribution of contact investigation to overall case notification is likely to be dependent on coverage of eligible index cases and we did observe a significant correlation between increased index case coverage and relative contribution to notification. Only three of thirteen projects with data systematically performed contact investigation on more than 70% of all index cases. This highlights an important area for potential expansion and further gains, provided program capacity and adequate resources are available. A systematic analysis of gaps and options using a public health framework approach can guide necessary steps for improvement.[30]

It is important to note that, although a successful screening program may detect and confirm many cases of TB, these are not necessarily all going to be cases who would have failed to seek care in the absence of contact investigation[31]. To justify the additional resources required, it is crucial that the effect of contact investigation on case notification and ultimately on disease transmission be studied. The interventions within this study showed an average increase in overall case notification of 26% (ranging from no increase to +100%).[11] However, the exact contribution of contact investigation to this increase could not be established as for most of the projects contact investigation was just one of several strategies adopted to improve case detection.

The long term effect of contact investigation may depend not only on increased numbers notified but also on its potential to find cases before they transmit the disease, at a stage when the symptoms are still limited and have not prompted individuals to seek care. Although our study does not provide direct evidence of this latter effect, the significantly higher percentage yield of active TB found in the projects that screened all contacts, irrespective of having symptoms, may be considered suggestive of such a potential. The full potential effect of contact investigation on case finding and transmission can only be assessed through randomized controlled trials or step wedge introduction of this intervention in a controlled setting[5]. To inform future policy, studies should include a detailed cost analysis and valid estimates of cost per additional case found. The identification of suitable locations to implement such a controlled study is problematic, since contact investigation is recommended by WHO in all settings[9] and has been included in national policies globally. Slow implementation of these policies[17] may still provide study opportunities that should be seized and funded.

Our study has some limitations. We have only aggregate data by project and no patient level data. Due to the analytical methods we used, the results are potentially biased towards the bigger studies; while a sensitivity analysis did not reveal a substantial bias, more research is needed to confirm our findings. The relatively small number of study settings and the wide variety of implementation practices, limited our ability to analyze the influence of each factor individually and influencing factors other than those in this study cannot be ruled out. The heterogeneity in context calls for caution to be used when interpreting the pooled screening yields; these yields cannot be generalized across all contexts.

Most projects used a similar definition for a household contact, but living and sleeping arrangements within households are likely to have differed across the projects. The effect of these variations on transmission within households can only be assessed by more detailed future studies using household level data.

The lack of availability of culture to confirm TB is an important limitation on the interpretation of the results. Despite the high specificity of SSM[25] we cannot exclude the possibility that some of the cases were diagnosed erroneously, particularly in the projects where all contacts were tested, irrespective of the presence of symptoms. If more sensitive screening algorithms are introduced, confirmation using a test with higher specificity should be considered. Furthermore, in our analysis we used the algorithms as reported by the project coordinators. Project staff may not in all cases have applied these consistently throughout the interventions. This potential loss of loyalty to protocol limits the strength of conclusion on the efficacy of a chosen algorithm. However the results reported here do represent their performance when implemented under general program conditions.

The contribution of HIV infection to tuberculosis transmission among household contacts—as reported by some authors[32;33]—could not be assessed, as HIV infection rates among index cases and contacts were not reported. Similarly, an association with the age of contacts, shown to be of relevance in other studies,[34] could not be evaluated, nor could we analyze the effect of drug resistance patterns.

## Conclusions

Our study shows that contact investigation can be a way to identify an important number of individuals with active disease, and has the potential for reducing the severity of the disease and decreasing its transmission by identifying cases at an earlier stage. However, given resource implications and the burden on overall service capacity, programs should carefully weigh the expected benefits against screening costs. The yield of cases found through contact investigation varies and is influenced by screening and testing algorithms such as the definition of index case and decisions on who to include for testing. Our data suggest that the use of more inclusive testing criteria for household contacts may yield more confirmed cases among contacts. Furthermore our data support current guidance to prioritize contact investigation on SS+ index cases. More rigorous studies are still needed to shape guidance on the protocols to be used across a variety of contexts.

Finally, although many countries have included contact investigation as part of their TB control strategy, we observed that it was generally not systematically implemented, underlining the need for additional resources to support methodical implementation of this WHO recommended policy.

## Supporting Information

S1 PRISMA Checklist (DOC)

S1 Table Overview screening data. (PDF)

S2 Table Sensitivity analysis by exclusion of five biggest studies. (PDF)

## References

[1] World Health Organization. Global tuberculosis report 2013 Geneva, Switzerland: WHO; 2013.

[2] Golub JE, Mohan CI, Comstock GW, Chaisson RE. Active case finding of tuberculosis: historical perspective and future prospects. Int J Tuberc Lung Dis 2005;9(11):1183–203. 16333924 PMC4472641

[3] Lönnroth K, Upplekar M, Blanc L. Early detection of Tuberculosis an overview of approaches, guidelines and tools World Health Organization; 2011.

[4] Lönnroth K, Corbett E, Golub J, Godfrey-Faussett P, Uplekar M, Weil D et al Systematic screening for active tuberculosis: rationale, definitions and key considerations. Int J Tuberc Lung Dis 2013;17(3):289–98. 10.5588/ijtld.12.0797 23407219

[5] Fox GJ, Dobler CC, Marks GB. Active case finding in contacts of people with tuberculosis. Cochrane Database Syst Rev 2011;(9):CD008477. 10.1002/14651858.CD008477.pub2 PMC6532613 21901723

[6] Tuberculosis Coalition for Technical Assistance. International Standards for Tuberculosis Care (ISTC). The Hague: Tuberculosis Coalition for Technical Assistance; 2006.

[7] Fox GJ, Barry SE, Britton WJ, Marks GB. Contact investigation for tuberculosis: a systematic review and meta-analysis. Eur Respir J 2013;41(1):140–56. 10.1183/09031936.00070812 22936710 PMC3533588

[8] Morrison J, Pai M, Hopewell PC. Tuberculosis and latent tuberculosis infection in close contacts of people with pulmonary tuberculosis in low-income and middle-income countries: a systematic review and meta-analysis. Lancet Infect Dis 2008;8(6):359–68 10.1016/S1473-3099(08)70071-9 18450516

[9] World Health Organization. Recommendations for investigating contacts of persons with infectious tuberculosis in low- and middle-income countries Geneva: WHO; 2013. 24404639

[10] Kranzer K, Afnan-Holmes H, Tomlin K, Golub JE, Shapiro AE, Schaap A et al The benefits to communities and individuals of screening for active tuberculosis disease: a systematic review. Int J Tuberc Lung Dis 2013;17(4):432–46. 10.5588/ijtld.12.0743 23485377

[11] Creswell J, Sahu S, Blok L, Bakker MI, Stevens R, Ditiu L. A multi-site evaluation of innovative approaches to increase tuberculosis case notification: summary results. PLoS One 2014;9(4):e94465 10.1371/journal.pone.0094465 24722399 PMC3983196

[12] Rembold CM. Number needed to screen: development of a statistic for disease screening. BMJ 1998;317(7154):307–12. 9685274 10.1136/bmj.317.7154.307 PMC28622

[13] Hamza TH, van Houwelingen HC, Stijnen T. The binomial distribution of meta-analysis was preferred to model within-study variability. J Clin Epidemiol 2008;61(1):41–51. 18083461 10.1016/j.jclinepi.2007.03.016

[14] World Health Organization. Country Health Statistics website. Available: http://www.who.int/countries/en/. Accessed 23–9–2014.

[15] Shah SA, Qayyum S, Abro R, Baig S, Creswell J. Active contact investigation and treatment support: an integrated approach in rural and urban Sindh, Pakistan. Int J Tuberc Lung Dis 2013;17(12):1569–74. 10.5588/ijtld.13.0169 24200270

[16] Mitchell EMH, Shapiro A, Golub J, Kranzer K, Portocarrero AV, Najlis CA et al. Acceptability of TB screening Among At-Risk and Vulnerable Groups: A systematic Qualitative/Quantitative Literature Metasynthesis. 2012; Available: http://www.who.int/tb/tbscreening/en/. Accessed 23 Dec 2014.

[17] Hwang TJ, Ottmani S, Uplekar M. A rapid assessment of prevailing policies on tuberculosis contact investigation. Int J Tuberc Lung Dis 2011;15(12):1620–3. 10.5588/ijtld.11.0222 22118168

[18] Shapiro AE, Variava E, Rakgokong MH, Moodley N, Luke B, Salimi S et al Community-based targeted case finding for tuberculosis and HIV in household contacts of patients with tuberculosis in South Africa. Am J Respir Crit Care Med 2012;185(10):1110–6. 10.1164/rccm.201111-1941OC 22427532 PMC5448579

[19] van't Hoog AH, Laserson KF, Githui WA, Meme HK, Agaya JA, Odeny LO et al High prevalence of pulmonary tuberculosis and inadequate case finding in rural western Kenya. Am J Respir Crit Care Med 2011;183(9):1245–53. 10.1164/rccm.201008-1269OC 21239690

[20] Kranzer K, Houben RM, Glynn JR, Bekker LG, Wood R, Lawn SD. Yield of HIV-associated tuberculosis during intensified case finding in resource-limited settings: a systematic review and meta-analysis. Lancet Infect Dis 2010;10(2):93–102. 10.1016/S1473-3099(09)70326-3 20113978 PMC3136203

[21] Hoa NB, Cobelens FGJ, Sy DN, Nhung NV, Borgdorff MW, Tiemersma EW. Yield of interview screening and chest X-ray abnormalities in a tuberculosis prevalence survey. Int J Tuberc Lung Dis 2012;16(6):762–7. 10.5588/ijtld.11.0581 22507287

[22] van't Hoog AH, Meme HK, Laserson KF, Agaya JA, Muchiri BG, Githui WA et al Screening strategies for tuberculosis prevalence surveys: the value of chest radiography and symptoms. PLoS One 2012;7(7):e38691 10.1371/journal.pone.0038691 22792158 PMC3391193

[23] van't Hoog AH, Langendam MW, Mitchell E, Cobelens FG, Sinclair D, Leeflang MMG et al. A systematic review of the sensitivity and specificity of symptom- and chest-radiography screening for active pulmonary tuberculosis in HIV-negative persons and persons with unknown HIV status. Available: http://www.who.int/tb/Review2Accuracyofscreeningtests.pdf?ua=1. Accessed 23 December 2014

[24] Nishikiori N, Van WC. Target prioritization and strategy selection for active case-finding of pulmonary tuberculosis: a tool to support country-level project planning. BMC Public Health 2013;13:97 10.1186/1471-2458-13-97 23374118 PMC3602078

[25] Steingart KR, Ng V, Henry M, Hopewell PC, Ramsay A, Cunningham J et al Sputum processing methods to improve the sensitivity of smear microscopy for tuberculosis: a systematic review. Lancet Infect Dis 2006;6(10):664–74. 17008175 10.1016/S1473-3099(06)70602-8

[26] van't Hoog AH, Onozaki I, Lonnroth K. Choosing algorithms for TB screening: a modelling study to compare yield, predictive value and diagnostic burden. BMC Infect Dis 2014;14(1):532. 25326816 10.1186/1471-2334-14-532 PMC4287425

[27] Creswell J, Codlin A, Andre E, Micek M, Bedru A, Carter E et al Results from early programmatic implementation of Xpert MTB/RIF testing in nine countries. BMC Infectious Diseases 2014;14(1):2 24383553 10.1186/1471-2334-14-2 PMC3898850

[28] World Health Organization. Monitoring of MTB/RIF roll-out. Available: http://www.who.int/tb/laboratory/mtbrifrollout/en/index.html. Accessed 3 July 2014.

[29] Ottmani S, Zignol M, Bencheikh N, Laasri L, Blanc L, Mahjour J. TB contact investigations: 12 years of experience in the National TB Programme, Morocco 1993–2004. La Revue de Santé de la Méditerranée orientale 2009;15(3):494–503. 19731765

[30] Hill PC, Rutherford ME, Audas R, van CR, Graham SM. Closing the policy-practice gap in the management of child contacts of tuberculosis cases in developing countries. PLoS Med 2011;8(10):e1001105 10.1371/journal.pmed.1001105 22022234 PMC3191150

[31] Blok L, Creswell J, Stevens R, Brouwer M, Ramis O, Weil O et al A pragmatic approach to measuring, monitoring and evaluating interventions for improved tuberculosis case detection. Int Health 2014 September;6(3):181–8. 10.1093/inthealth/ihu055 25100402 PMC4153747

[32] Kenyon TA, Creek T, Laserson K, Makhoa M, Chimidza N, Mwasekaga M et al Risk factors for transmission of Mycobacterium tuberculosis from HIV-infected tuberculosis patients, Botswana. Int J Tuberc Lung Dis 2002;6(10):843–50. 12365569

[33] Elliott AM, Hayes RJ, Halwiindi B, Luo N, Tembo G, Pobee JO et al The impact of HIV on infectiousness of pulmonary tuberculosis: a community study in Zambia. AIDS 1993;7(7):981–7. 8357557 10.1097/00002030-199307000-00012

[34] World Health Organization Stop TB Partnership Childhood TB Subgroup. Chapter 4: childhood contact screening and management. Int J Tuberc Lung Dis 2007;11(1):12–5. 17217124

