# Supplementary material for: Comparative Meta-Analysis of Tuberculosis Contact Investigation Interventions in Eleven High Burden Countries
Source: PLoS One. 2015 Mar 26;10(3):e0119822. doi: 10.1371/journal.pone.0119822 (PMC4374904; doi:10.1371/journal.pone.0119822)
Supplement: S1 Table — (PDF) [file pone.0119822.s002.pdf]

Screening data

	Index cases			Contacts identified screened and tested									cases confirmed among contacts							
proj_id	Eligible Index cases†	CI done	% coverage	Iden-tified	Screen-ed	% coverage	Symptom atic	Tested (sputum)	% coverage		Full screened contacts		SS+	% SS+ /screen	95% CI	All TB	% All TB /screen	95% CI	% notified SS+ found through CI	
Afghanistan-1	2382				7232			1480					136	1.9%	(1.6-2.2%)				5.7%	(4.8-6.6%)
Afghanistan-2	4777	2894	60.6%		19383			6782					210	1.1%	(0.9-1.2%)	307	1.6%	(1.4-1.8%)	4.4%	(3.8-5.0%)
DRC-1	2610				5814			715					166	2.9%	(2.4-3.3%)				6.4%	(5.4-7.3%)
DRC-2	6773	4528	66.9%	25264	18384	72.8%	3685	3428	93.0%		67.7%		322	1.8%	(1.6-1.9%)	397	2.2%	(1.9-2.4%)	4.8%	(5.0-6.2%)
DRC-3	6360	5831	91.7%	20672	16552	80.1%	2940	2719	92.5%		74.1%		300	1.8%	(1.6-2.0%)	464	2.8%	(2.6-3.1%)	4.7%	(5.2-6.5%)
DRC-4	6353	4346	68.4%	30951	13265	42.9%	1369	1236	90.3%		38.7%		127	1.0%	(0.8-1.1%)	178	1.3%	(1.1-1.5%)	2.0%	(2.2-3.1%)
Ethiopia-1	687				490						0.0%		11	2.2%	(0.9-3.6%)				1.6%	(0.7-2.5%)
Ethiopia-2	5090	3499	68.7%	8005	8005	100.0%	1949	1290	66.2%		66.2%		62	0.8%	(0.6-1.0%)	69	0.9%	(0.7-1.1%)	1.2%	(0.9-1.5%)
Kenya-1	3121	151	4.8%		742			147					3	0.4%	(0.0-0.9%)				0.1%	(0.0-0.2%)
Kenya-2	12780	2860	22.4%		6274		3482	2246	64.5%				87	1.4%	(1.1-1.7%)	133	2.1%	(1.8-2.5%)	0.7%	(0.5-0.8%)
Lao rep	2179	61	2.8%		316			26					1	0.3%	(0.0-0.9%)				0.0%	(0.0-0.1%)
Nepal	4338				6596		2708	2529	93.4%		0.0%		68	1.0%	(0.8-1.3%)				1.6%	(1.2-1.9%)
Nigeria	2532				387		86	58	67.4%		0.0%		9	2.3%	(0.8-3.8%)				0.4%	(0.1-0.6%)
Pakistan-1	3608	3037	84.2%		19191		3478	2160	62.1%				490	2.6%	(2.3-2.7%)	538	2.8%	(2.5-3.0%)	13.6%	(12.5-14.7%)
Pakistan-2	3230	481	14.9%	3704	3111	84.0%	316	43	13.6%		11.4%		4	0.1%	(0.0-0.3%)	19	0.6%	(0.3-0.9%)	0.1%	(0.0-0.6%)
Uganda-1	2041	1077	52.8%		4638					(*)	0.0%		287	6.2%	(5.5-6.9%)				14.1%	(12.6-15.6%)
Uganda-2	2259	308	13.6%	882	573	65.0%		483	84.3%	(*)	54.8%	(*)	32	5.6%	(3.7-7.5%)				1.4%	(0.9-1.9%)
Yemen		1030		5059	3200	63.3%	1356	2093	65.4%	(*)	41.4%	(*)	123	3.8%	(3.2-4.5%)	152	4.8%	(4.0-5.5%)		
Zimbabwe	2346	1313	56.0%		4899			210					60	1.2%	(0.9-1.5%)				2.6%	(1.9-3.2%)
					139052								2498							

† Number of eligible index cases equals the number of SS+ cases in the population notified during the project period , with exception of

Note: empty cells indicate data not available
