# Supplementary material for: Comparative Meta-Analysis of Tuberculosis Contact Investigation Interventions in Eleven High Burden Countries
Source: PLoS One. 2015 Mar 26;10(3):e0119822. doi: 10.1371/journal.pone.0119822 (PMC4374904; doi:10.1371/journal.pone.0119822)
Supplement: S2 Table — (PDF) [file pone.0119822.s003.pdf]

Table S2. Sensitivity analysis (exclusion of 5 biggest studies) for the association between programme design or contextual factors and percentage yield of contact tracing

				Multi variate model (N=19)			Multi variate model (N=14) Excluding 5 biggest projects(‡)		
		N projects	N screened	aOR	(95% CI)	p-value (*)	aOR	(95% CI)	p-value (*)
**Program design and implementation**									
Definition index case									
- index includes children	reference	15	8677	dropped in backward elimination			dropped in backward elimination		
- index does not include children		4	130375						
- SS+ index cases only	reference	15	87740	1			1		
- SS+ and (SS- and/or EPTB) index cases		4	51312	**0.31**	**(0.15-0.62)**	**0.001**	**0.10**	**(0.03-0.30)**	**<0.001**
Definition Suspected TB (people to be tested)		19	139052			**<0.001**			**0.003**
- Contact with cough at least 2 weeks	reference	6	41666	1			1		
- Contact with any TB related symptoms		10	88975	1.71	(0.94-3.13)	0.080	1.05	(0.72-1.86)	0.558
- Any HH contact irrespective of symptoms		3	8411	**6.90**	**(3.42-13.93)**	**<0.001**	**5.44**	**(3.20-9.25)**	**<0.001**
**Context and setting**									
Background TB prevalence (unit of increase: 100/100,000 pop)		19	139052	1.22	(0.99-1.50)	0.057	1.17	(0.98-1.39)	0.076
Setting									
Rural	reference	12	95039	1			1		
Urban/mixed		7	44013	**0.53**	**(0.31-0.90)**	**0.018**	0.67	(0.43-1.03)	0.067

* Wald test of significance of effect; LLR test of significance of variable in the model

‡ Excluded are Afghanistan-2, DRC-2, DRC-3, DRC-4, and Pakistan-1
